# TNF-*α*-induced LRG1 promotes angiogenesis and mesenchymal stem cell migration in the subchondral bone during osteoarthritis

**DOI:** 10.1038/cddis.2017.129

**Published:** 2017-03-30

**Authors:** Yiyun Wang, Jiajia Xu, Xudong Zhang, Chuandong Wang, Yan Huang, Kerong Dai, Xiaoling Zhang

**Affiliations:** 1 The Key Laboratory of Stem Cell Biology, Institute of Health Sciences, Shanghai Institutes for Biological Sciences (SIBS), Chinese Academy of Sciences (CAS); University of Chinese Academy of Sciences, Shanghai 200031, China; 2 Shanghai Key Laboratory of Orthopaedic Implant, Department of Orthopaedic Surgery, Shanghai Ninth People's Hospital, Shanghai Jiao Tong University School of Medicine (SJTUSM), Shanghai 200011, China

## Abstract

The incomplete understanding of aberrant neovascularization, which contributes to osteoarthritis suggests that additional modulators have yet to be identified. Our objective was to identify the role of Leucine-rich-alpha-2-glycoprotein1 (LRG1), a new regulator of pathogenic angiogenesis, in osteoarthritis progression and to develop effective treatment strategies. In this study, immunohistochemistry showed that LRG1 was increased in the subchondral bone and articular cartilage in anterior cruciate ligament transection (ACLT) mice. Further studies were focused on the role of LRG1 in osteoarthritis. Results showed that LRG1 promoted angiogenesis and mesenchymal stem cells (MSC) migration, which contribute to aberrant bone formation in the subchondral bone. Moreover, tumor necrosis factor-*α* (TNF-*α*), not interleukin-1*β* (IL-1*β*), IL-6 or IL-17, induced the LRG1 expression in human umbilical vein endothelial cells and this effect was inhibited by p38 mitogen-activated protein kinase or NF-*κ*B inhibitor. Notably, inhibition of TNF-*α* and LRG1 activity by Lenalidomide, an inhibitor of TNF-*α* production, in ACLT mice attenuated degeneration of osteoarthritis articular cartilage. This study shows that TNF-*α* is the predominant proinflammatory cytokine that induces the secretion of LRG1. LRG1 contributes to angiogenesis-coupled *de novo* bone formation by increasing angiogenesis and recruiting MSCs in the subchondral bone of osteoarthritis joints. Inhibition of TNF-*α* and LRG1 by Lenalidomide could be a potential therapeutic approach.

Osteoarthritis (OA), a degenerative joint disorder, is the leading cause of pain and disability in the aging population. OA has been projected to affect 67 million people by 2030 in the United States, resulting in adverse effects on individuals and health system burden.^1^ Unfortunately, although many defined factors (such as biomechanical, metabolic, inflammatory or genetic factors) are associated with OA, the exact pathogenesis remains unclear.^2^ Therefore, the lack of disease-modifying treatment necessitates joint replacement for some severely affected patients.^3^

Angiogenesis contributes to structural damage and pain in OA progression.^4, 5^ Healthy articular cartilage is without vessels and nerves, but during OA progression, cells within the subchondral bone express angiogenic factors, such as vascular endothelial growth factor (VEGF) and platelet-derived growth factor subunit B (PDGFB), to increase endothelial cell proliferation and vascular densities. This results in vessels and channels invading from the subchondral bone into the cartilage zones to facilitate endochondral ossification.^6, 7^ The increase of angiogenesis recruits more lymphocytes, macrophages and other inflammatory cells.^8^ The subchondral bone, a structural girder and shock absorber, provides mechanical support for the overlying articular cartilage.^9, 10^ Therefore, the changes in the subchondral bone, which result in the alteration of the subchondral bone's thickness and flexibility, contribute to cartilage lesions and aggravate pain.^3, 6^ The vessels are large in the subchondral bone of anterior cruciate ligament transection (ACLT) mice.^3^ The aberrant bone formation through increased nestin-positive mesenchymal stem cells (MSCs) in the subchondral bone is coupled with angiogenesis.^3^ However, the incomplete understanding of aberrant neovascularization, which contributes to articular cartilage degeneration during osteoarthritis progression suggests that additional modulators have yet to be identified.

Leucine-rich-alpha-2-glycoprotein1 (LRG1) is a new regulator of pathogenic angiogenesis and a novel oncogene-associated protein.^11, 12^ Reports showed that LRG1 has an important role in epithelial–mesenchymal transition and angiogenesis in colorectal cancer; and glioma cell invasion, migration and angiogenesis promotion in the damaged retina.^11, 12, 13^ Reports showed that LRG1 promotes angiogenesis by modulating endothelial transforming growth factor b1 (TGF-*β*) signaling. In the angiogenesis condition, LRG1 modulates TGF-*β* to activate smad1/5 phosphorylation.^11^ LRG1 is a promising therapeutic target for pathogenic angiogenesis-associated disease, such as ocular disease, cancer and atherosclerosis,^11^ but it remains unclear whether LRG1 is increased and plays a role in OA or not.

Tumor necrosis factor-*α* (TNF-*α*) is one of the main proinflammatory cytokines involved in OA pathogenesis. TNF-*α* stimulates the release of matrix metalloproteinase-1 (MMP-1), MMP-3 and MMP13 and suppresses proteoglycan and type II collagen synthesis in OA.^2^ TNF-*α* is also a stimulus for the local release of angiogenic substances, such as VEGF, basic fibroblast growth factor (bFGF) and PDGFB, which promote the proliferation and migration of endothelial cells.^8, 14^ Whether TNF-*α* affects LRG1 secretion is not known. The small molecule Lenalidomide (LEN) is an inhibitor of TNF-*α* production.^15^ LEN has shown therapeutic promises in clinical trials for myeloma in consideration of its antiangiogenic and anti-inflammatory effects.^16, 17, 18^ Other researches have also focused on its effects in solid tumors,^19, 20^ but little is known about its treatment effects on OA.

In this study, the role of LRG1 in the subchondral bone and articular cartilage during OA progression was investigated. We found that LRG1 was elevated in the subchondral bone and articular cartilage in a mouse OA model. LRG1 promoted the angiogenesis of endothelial cells and the migration of MSC coupling with bone formation. Furthermore, we demonstrated that TNF-*α* stimulated LRG1 secretion and found the possible signaling pathway involved. Finally, we found that the inhibition of TNF-*α* production by LEN reduced LRG1 expression, attenuated the pathological changes of subchondral bone and alleviated the degeneration of articular cartilage compared with vehicle-treated groups in the OA models. Our experimental data provide the following: (i) novel molecular mechanisms of OA, suggesting the role of LRG1 that is induced by TNF-*α* and (ii) a novel potential preventive treatment drug for OA.

## Results

### The expression of LRG1 in the subchondral bone and articular cartilage of ACLT mice

**Figure 1:**
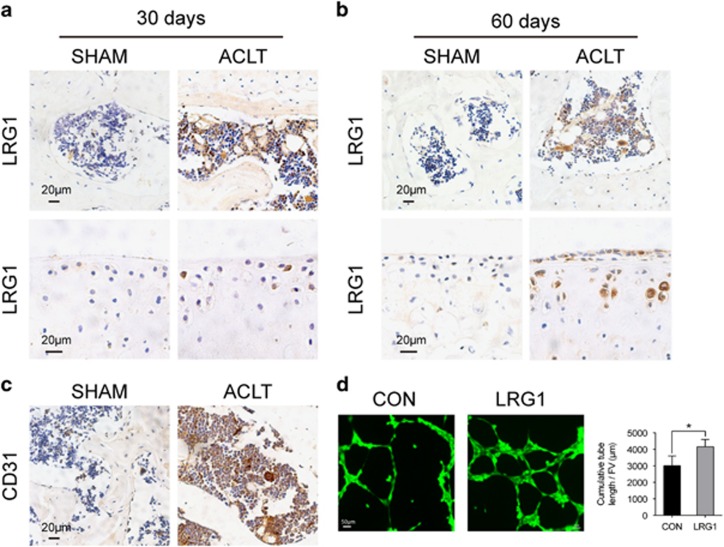
LRG1 was upregulated in the subchondral bone and articular cartilage and associated with angiogenesis in ACLT mice. (**a** and **b**) Immunohistochemical analysis results of LRG1 in mouse tibial subchondral bone (top) and articular cartilage (bottom) collected 30 days after ACLT surgery (**a**) and collected 60 days after ACLT surgery (**b**); *n*=5 per group. Scale bars, 20 *μ*m. (**c**) Immunohistochemical analysis of CD31 in mouse tibial subchondral bone; *n*=5 per group. Scale bars, 20 *μ*m. (**d**) Matrigel tube-formation assay images (left) and quantitative analysis of cumulative tube length (right). Scale bar, 50 *μ*m. Values are given as means±S.D. (three independent experiments), *P<0.05

Angiogenesis is essential in the progression of OA, and we wondered whether LRG1, a new regulator of pathogenic angiogenesis, has an important role in OA. We examined LRG1 expression by immunohistochemistry. We found that LRG1 was upregulated in the subchondral bone in ACLT mice compared with sham-operated mice at 30 and 60 days after operation (Figures 1a and b). We also found that LRG1 significantly upregulated in the articular cartilage 60 days after operation and slightly upregulated 30 days after operation (Figures 1a and b) The number of CD31-positive endothelial cells was larger in the subchondral bone of ACLT mice (Figure 1c). The effectiveness of promoting angiogenesis was verified by HUVEC tube-formation assay *in vitro*. The LRG1-adding groups showed an enhanced ability to form capillary-like structures compared with vehicle groups (Figure 1d). These results suggested that LRG1 promoted angiogenesis in the subchondral bone of ACLT mice.

### The effect of LRG1 on MSC migration

**Figure 2:**
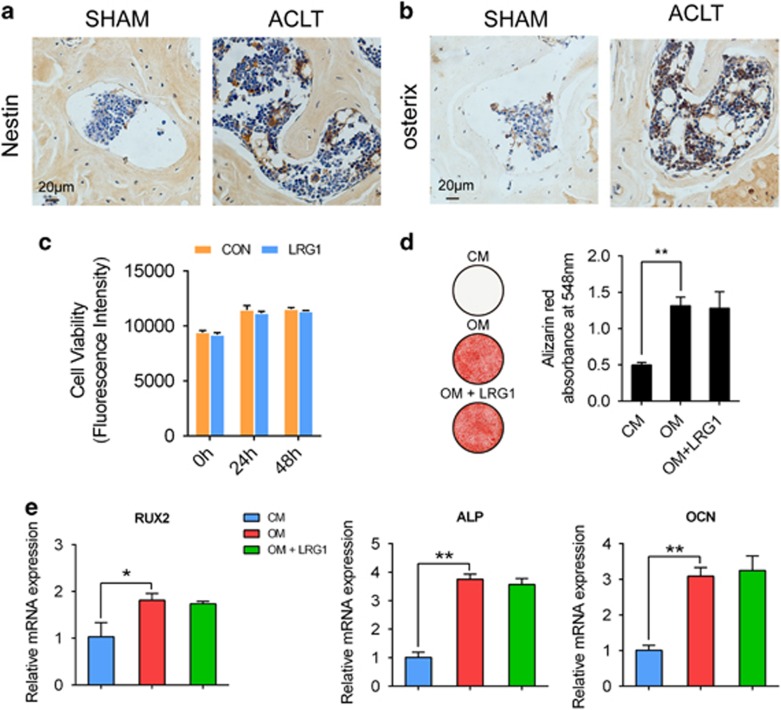
LRG1 had no direct effect on osteogenesis. (**a** and **b**) Immunohistochemical analysis of nestin (**a**) and osterix (**b**) in mouse tibial subchondral bone collected 30 days after ACLT surgery, *n*=5 per group. Scale bars, 20 *μ*m. (**c**) The cell proliferation was determined in hBMMSCs by PrestoBlue. (**d**) Alizarin Red S staining (left) of cultured hBMMSCs after treatment with osteogenic medium plus LRG1. Quantitative of Alizarin Red S staining showed the levels of mineralization (right). CM, control medium; OM, osteogenesis medium; OM+LRG1, osteogenesis medium plus LRG1. (**e**) Relative expression levels of osteoblast markers in hBMMSCs indicated in **d** were quantified by RT-PCR. All values are given as means±S.D. (three independent experiments); *P<0.05, **P<0.01

**Figure 3:**
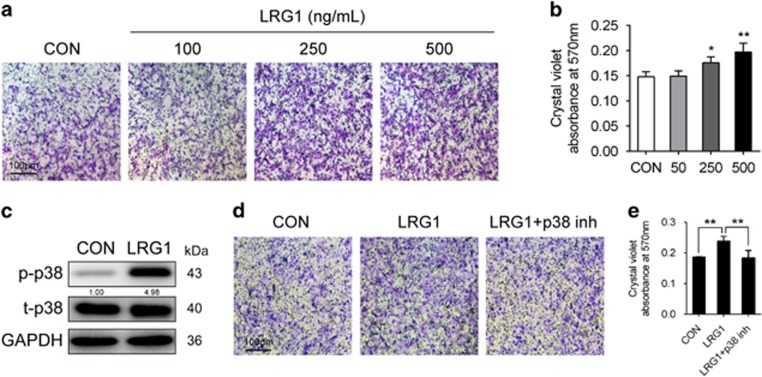
LRG1 induced hBMMSCs migration. (**a** and **b**) Transwell assay images (**a**) and quantitative analysis (**b**) for the migration of hBMMSCs by using mediums plus different concentrations of LRG1. Scale bar, 100 *μ*m. (**c**) Western blots of the p38 phosphorylation in hBMMSCs treated with LRG1 for 15 min. The relative expression levels of protein are shown at the bottom of the bands as normalized by the total p38 level. (**d** and **e**) Transwell assay images (**d**) and quantitative analysis (**e**) for the migration of hBMMSCs that were preincubated with vehicle or p38 inhibitor (inh p38) by using indicated mediums. Scale bar, 100 *μ*m. All values are given as means±S.D. (three independent experiments); *P<0.05, **P<0.01

We found that immunostaining for nestin revealed a significantly larger number of nestin-positive cells in the subchondral bone of ACLT mice compared with sham-operated groups (Figure 2a). Nestin is supposed to be expressed primarily in adult bone marrow MSCs.^21, 22^ Once committed to the osteoblast lineage, MSCs express osterix that is essential for osteoblastogenesis. The number of osterix-positive cells was higher in the subchondral bone of ACLT mice compared with sham-operated controls (Figure 2b), which indicated that the nestin-positive MSCs might participate in the *de novo* bone formation through osteoblastic differentiation. Thus, we explored whether LRG1 contributes to the increased nestin-positive MSCs in ACLT mice. To confirm this, we cultured hBMMSCs exposed to LRG1 to analyze cell proliferation, but LRG1-treated hBMMSCs had no significant difference with the controls in proliferation (Figure 2c). Then, we induced osteogenic differentiation of the human bone marrow mesenchymal stem cells (hBMMSCs) to find whether LRG1 promotes osteogenesis or not. However, LRG1 exerted no direct promotion effects on osteogenesis (Figures 2d and e). However, LRG1 induced significant hBMMSC migration relative to the control (Figures 3a and b). Mitogen-activated protein kinases (MAPKs) are known to have important roles in cell migration.^23, 24^ We assessed their roles in LRG1-induced hBMMSC migration. Western blot analysis results showed that LRG1 significantly induced phosphorylation of p38 in hBMMSCs as shown in Figure 3c. To confirm that LRG1-induced migration was mediated through p38 pathway, the phosphorylation of p38 was blocked by p38 MAPK inhibitor in the Transwell assays. The inhibition of p38 pathway was sufficient to block LRG1-induced migration of hBMMSCs (Figures 3d and e). The results indicated that instead of promoting osteogenesis directly, LRG1 promoted hBMMSC migration through p38 signaling to contribute in the osteogenesis.

### The effect of TNF-*α* on LRG1 secretion

**Figure 4:**
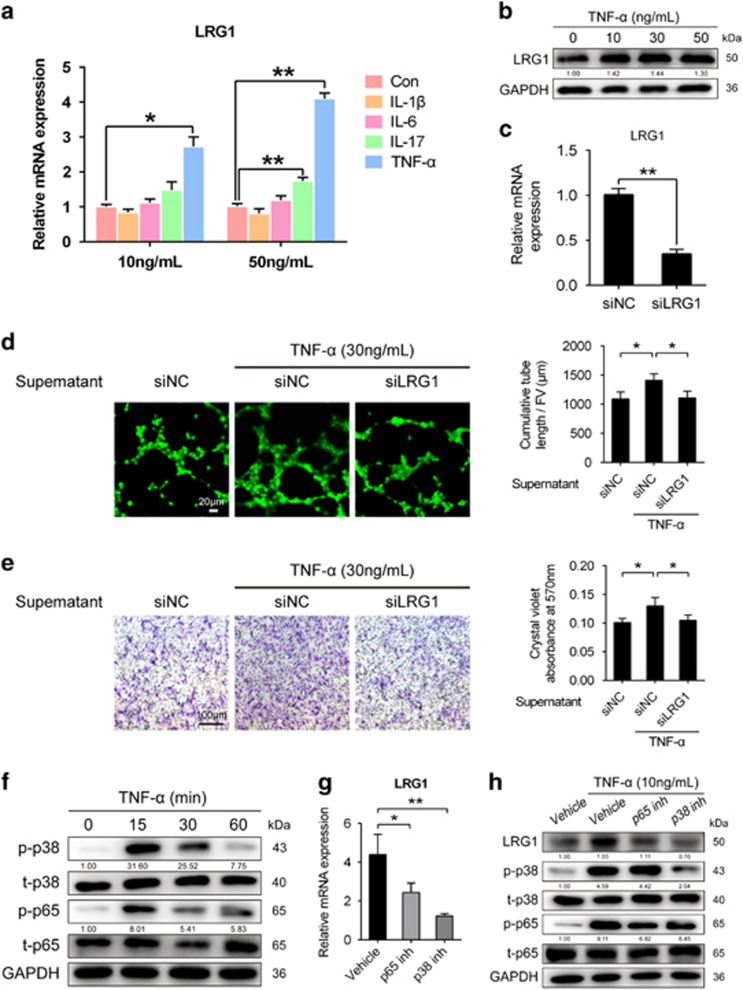
TNF-*α* induced LRG1 secretion in HUVECs through p38 and p65 signaling. (**a**) RT-PCR analysis of LRG1 in HUVECs, stimulated with IL-1*β*, TNF-*α*, IL-6 and IL-17 (10 or 50 ng/ml). (**b**) Western blot analysis of LRG1 in HUVECs stimulated with TNF-*α* in different concentrations. The relative expression levels of protein are shown at the bottom of the bands as normalized by the GAPDH level. (**c**) Knockdown of LRG1 expression in HUVECs by siRNA. (**d**) Matrigel tube-formation assay of HUVECs after treatment with supernatants, which were collected from siLRG1 or siNC transfected HUVEC cultures in the presence of TNF-*α*. Images (left) and quantitative analysis of cumulative tube length (right). Scale bar, 20 *μ*m. (**e**) Transwell assays of hBMMSCs after treatment with supernatants, which were collected from siLRG1 or siNC transfected HUVEC cultures in the presence of TNF-*α*. Images (left) and quantitative analysis (right). Scale bar, 100 *μ*m. (**f**) Western blot analysis of the phosphorylation of p38 and p65 in HUVECs treated with TNF-*α* at different times. The relative expression levels of protein are shown at the bottom of the bands as normalized by the total p38 and p65 levels, respectively. (**g** and **h**) RT-PCR (**g**) or western blot analysis of LRG1 and phosphorylation of p38 and p65 (**h**) in HUVECs stimulated with TNF-*α* and pretreated or not with p38 inhibitor (p38 inh) and p65 inhibitor (p65 inh). The relative expression levels of protein are shown at the bottom of the bands. LRG1 level is normalized by the GAPDH level, and the phosphorylation of p38 and p65 levels are normalized by the total p38 and p65 levels, respectively. All values are given as means±S.D. (three independent experiments); *P<0.05, **P<0.01

Interleukin-1*β* (IL-1*β*), TNF-*α*, IL-6 and IL-17are considered to be the main proinflammatory cytokines involved in the pathophysiology of OA.^2^ These proinflammatory cytokines upregulate angiogenic factors to stimulate angiogenesis. Therefore, we investigated the influence of these proinflammatory cytokines on LRG1 secretion in human umbilical vein endothelial cells (HUVECs). To this end, we assessed the changes in the mRNA expression of LRG1 in HUVECs treated with recombinant IL-1*β*, TNF-*α*, IL-6 and IL-17 (10 or 50 ng/ml). Among the tested cytokines, TNF-*α*-treated groups significantly increased the expression of LRG1 in both dosages, whereas IL-1*β* and IL-6 had no obvious effects on LRG1 expression. IL-17 slightly upregulated LRG1 in 50 ng/ml not 10 ng/ml (Figure 4a). We further analyzed the levels of LRG1 protein in HUVECs treated with different dosages of recombinant TNF-*α* (10−50 ng/ml) by western blot. As expected, the TNF-*α* increased LRG1 expression (Figure 4b). To affirm that TNF-*α* induces the secretion of LRG1 in HUVECs to promote angiogenesis and MSC migration, we used siRNA to inhibit LRG1 expression in HUVECs (Figure 4c). We collected the supernatants of siLRG1 or siNC-treated HUVECs that were induced with TNF-*α*. Then, we used the supernatants to induce HUVEC tube formation and hBMMSC migration. The knockdown of LRG1 expression led to a significant decrease in HUVEC tube formation and hBMMSC migration (Figures 4d and e). The activation of MAPKs and NF-*κ*B is known to regulate TNF-*α*-induced gene expression. Western blot analysis showed that TNF-*α* induced phosphorylation of p38 and p65 in HUVECs (Figure 4f). To verify the involvement of these signaling in upregulated LRG1 responses to TNF-*α*, we pretreated HUVECs with the MAPKs or NF-*κ*B inhibitor independently. We found that p38 MAPK inhibitor and NF-*κ*B inhibitor reduced TNF-*α*-induced LRG1 mRNA expression (Figure 4g). Consistently, western blot analysis showed that TNF-*α* induced LRG1 protein level was reduced by p38 MAPK and NF-*κ*B inhibitors (Figure 4h). These data indicated that TNF-*α* induced the expression of LRG1 through p38 and NF-*κ*B signaling to promote angiogenesis and MSC migration.

### Inhibition of TNF-*α* and LRG1 by LEN in OA

**Figure 5:**
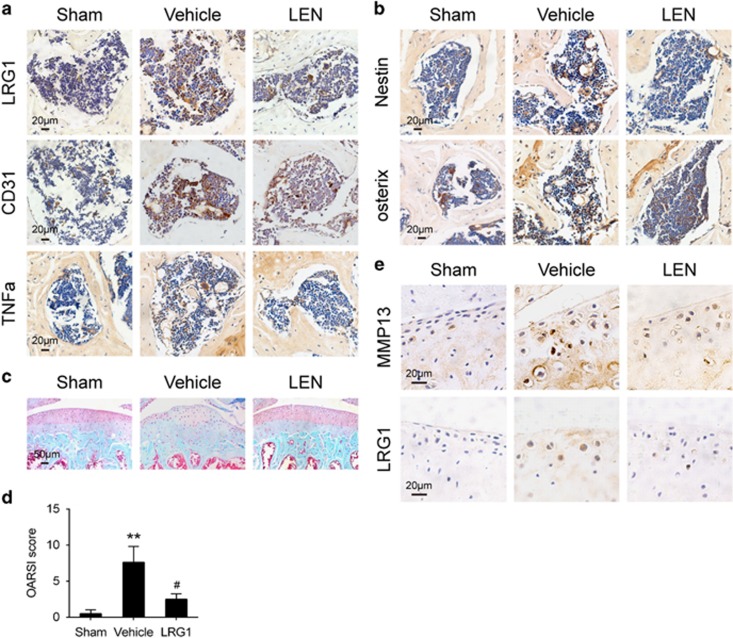
LEN, a TNF-*α* production inhibitor, reduced LRG1 expression, alleviated the changes in the subchondral bone, and attenuated articular cartilage degeneration in ACLT. (**a** and **b**) Immunohistochemical analysis of LRG1 (top, **a**), CD31 (middle, **a**), TNF-*α* (bottom, **a**), nestin (Top, **b**) and osterix (bottom, **b**) in tibial subchondral bone harvested 30 days after sham operation (sham), ACLT operation and treatment with vehicle (vehicle) or ACLT operation and treatment with LEN (LEN); *n*=5 per group. Scale bars, 20 *μ*m. (**c**) Safranin O and fast green staining in the articular cartilage of mice 60 days after surgery; *n*=5 per group. Scale bars, 50 *μ*m. (**d**) OARSI scores in **c**. (**e**) Immunohistochemical analysis of MMP13 (top) and LRG1 (bottom) in the articular cartilage of mice 30 days after surgery; *n*=5 per group. Scale bars, 20 *μ*m. All values are given as means±S.D. **P<0.01 compared with the sham-operated group; ^#^*P*<0.05 compared with the vehicle-treated ACLT group

LEN is an inhibitor of TNF-*α* production.^15^ We are interested in whether the inhibition of TNF-*α* secretion by LEN reduces LRG1 expression, and diminishes angiogenesis and aberrant bone formation to attenuates OA. So, we injected LEN (50 mg per kg body weight) intraperitoneally daily in ACLT mice. The immunostaining of the subchondral bone showed that TNF-*α* was higher in ACLT mice relative to sham-operated controls, and this effect was prevented by LEN treatment (Figure 5a). Consistently, the expression of LRG1 was significantly higher in the subchondral bone of ACLT mice relative to sham-operated controls, and this effect was inhibited by the injection of LEN (Figure 5a). Moreover, the number of CD31-positive endothelial progenitors was significantly larger in the subchondral bone of ACLT mice than that of sham-operated controls, and this effect was reduced by LEN treatment, suggesting reduced angiogenesis (Figure 5a).

We found by immunostaining that the increased number of nestin-positive MSCs in the subchondral bone of ACLT mice relative to sham-operated controls was reduced by the injection of LEN (Figure 5b). The number of osterix-positive osteoprogenitors was reduced by the injection of LEN in ACLT mice (Figure 5b), suggesting a decreased aberrant bone formation.

The degeneration of articular cartilage was attenuated by LEN administration for 2 months after surgery as shown in Figure 5c. A time point is usually used for the severity analysis in mechanical destabilized OA mice models.^25^ We quantified the protective effect of LEN on articular cartilage using the Osteoarthritis Research Society International (OARSI) system^26^ (Figure 5d).The immunostaining of the articular cartilage showed that the number of MMP13-positive chondrocytes was smaller in the LEN-treated than the vehicle-treated ACLT mice, reflecting the protection from articular cartilage degeneration (Figure 5e). The LRG1-positive chondrocytes were reduced in the LEN-treated than the vehicle-treated ACLT mice (Figure 5e). Collectively, LEN treatment attenuated the pathological changes in the subchondral bone and led to less degeneration of articular cartilage compared with vehicle-treated ACLT mice.

## Discussion

Angiogenesis is important in OA development. During OA, angiogenesis is increased in the subchondral bone, which leads to vascularization and lesion formation in the osteoarthritic cartilage.^3, 4, 6, 7^ The cells within the subchondral bone express angiogenic factors (such as VEGF and PDGFB) that increase endothelial cell proliferation and vascular densities in OA.^4, 6, 27^ Recently, a novel angiogenic factor, LRG1, was identified as a regulator of pathogenic angiogenesis.^11^ However, whether LRG1 has an important role in OA remains unclear. Thus, we hypothesized that LRG1 promoted the angiogenesis in the subchondral bone. Consistently, we found that LRG1 was upregulated in the subchondral bone marrow of ACLT mice. The chondrocytes also express some angiogenic factors that promote vessel and channel invasion from the subchondral bone into the cartilage zones.^4, 6, 27^ We further found that LRG1 was also upregulated in the articular cartilage of ACLT mice. Evidence has shown that blood vessels and the number of CD31+ endothelial progenitor cells in the subchondral bone were significantly higher in ACLT mice relative to sham mice.^3^ Consistently, we found that the number of CD31-positive cells in the subchondral bone marrow was larger in ACLT mice, revealing elevated angiogenesis. We confirmed that LRG1 enhanced the tube formation of HUVECs *in vitro* at different dosage. We found that LRG1 promoted tube-formation well at 500 ng/ml, which demonstrated the angiogenic effect. Therefore, these data suggest that LRG1 is elevated and promoted angiogenesis in OA.

OA is developing along with the accretion of mesenchymal progenitor cells in the joint tissues and synovial fluids.^3, 28, 29^ Consistently, mechanical destabilized joints exerted a large number of nestin-positive MSCs and osterix-positive osteoprogenitors in the subchondral bone marrow in ACLT mouse models in our study. The altered mechanical loading leads to aberrant bone formation by *in situ* commitment of osteoprogenitors rather than normal bone remodeling by osteoblasts and their progenitors at the resorption site on the bone surface.^3, 30^ In this study, we tested the ability of LRG1 to recruit hBMMSCs by Transwell assay. We found that LRG1 induced significant hBMMSCs migration. The enhanced migration was inhibited by the blockage of p38 signaling pathway. The ability of LRG1 to recruit MSCs may contribute to the *in situ* aberrant bone formation. The changes in the subchondral bone contribute to cartilage lesions and aggravate pain.^31^ Bone formation is often coupled with angiogenesis. The formation of new blood vessels is required for supplying nutrients, oxygen, growth factors and cytokines as required for bone tissue.^32, 33, 34^ Therefore, the elevated angiogenesis in the subchondral bone marrow in ACLT mice is convenient for the aberrant bone formation, and some angiogenic growth factors secreted by fibrovascular tissue stimulate the migration of MSCs.^35, 36^ Thus, we suggest that LRG1 has an important role in the angiogenesis coupling with aberrant bone formation by recruiting MSCs in the subchondral bone in OA.

Proinflammatory cytokines have important roles in the pathophysiology of OA, and many of them upregulate angiogenic factors to stimulate angiogenesis.^14, 37, 38, 39^ Thus, we tested the effects of main proinflammatory cytokines IL-1*β*, TNF-*α*, IL-6 and IL-17 on LRG1 expression in HUVECs. Our results showed that IL-1*β*, and IL-6 had no obvious effects on LRG1 expression and IL-17 at 50 ng/ml mildly upregulated LRG1. However, TNF-*α* significantly increased the expression of LRG1 in HUVECs. TNF-*α* has been shown to promote cell proliferation and angiogenesis.^40^ Thus, we hypothesized that TNF-*α* induces the secretion of LRG1 in HUVECs to promote angiogenesis and MSC migration. Consistently, the supernatants of TNF-*α*-stimulated HUVECs promoted angiogenesis and MSC migration, and this effect was prevented by the supernatants of siLRG1-treated HUVECs. MAPK and NF-*κ*B signaling pathway are typically involved in the downstream function of TNF-*α*.^40, 41^ Consistent with this, our study found that the inhibition of the kinases p38 and p65 reduced TNF-*α*-induced LRG1 secretion, suggesting that these molecules are implicated in the LRG1 expression in HUVECs. Results suggest that TNF-*α* is a proinflammatory cytokine of inducing LRG1 expression in OA pathogenesis.

The small molecule LEN is an inhibitor of TNF-*α* production.^15^ We hypothesized that the inhibition of TNF-*α* production by LEN reduced TNF-*α*-induced LRG1 secretion, and alleviated angiogenesis and aberrant bone formation to attenuate OA. To test our hypothesis, we injected LEN intraperitoneally daily in ACLT mice. We showed that LEN suppressed the expression of TNF-*α* and LRG1 and reduced the number of CD31-positive cells in the subchondral bone, suggesting diminished angiogenesis. It is known that the elevated vessels invade from the subchondral bone into the cartilage zones to facilitates endochondral ossification in OA.^6, 7^ The decreased angiogenesis probably alleviates the process. The reduction of nestin-positive MSCs and osterix-positive osteoprogenitors in the LEN-injected mice revealed less MSCs migration and aberrant bone formation. The decreased aberrant bone formation in the subchondral bone marrow probably alleviates the degeneration of articular cartilage. Consistently, it showed alleviated articular cartilage degeneration in LEN-treated mice. LEN has shown therapeutic promises in clinical trials for the treatment of multiple myeloma in consideration of its antiangiogenic and anti-inflammatory effects.^16, 17, 18, 19, 20^ Recently, some treatment strategies for OA are aimed at anti-TNF-*α* and anti-angiogenesis.^42, 43^ We expect that treatment with LEN is a potential approach for ameliorating OA.

**Figure 6:**
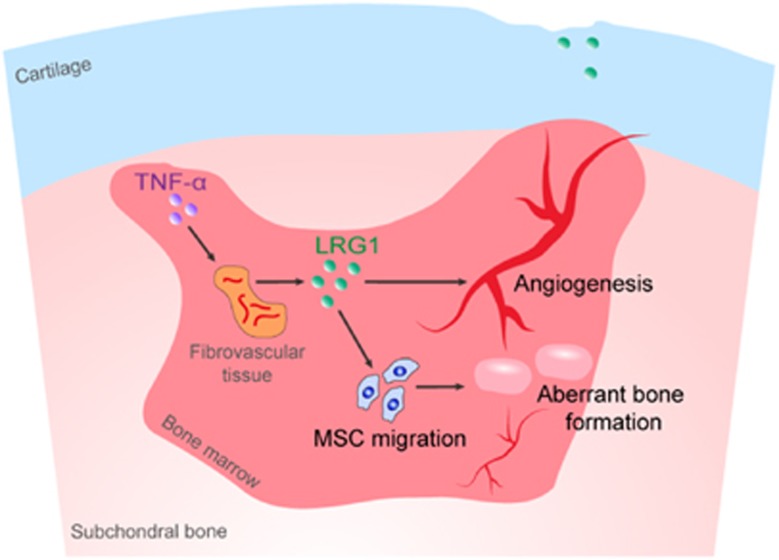
Proposed role of LRG1 in OA. The elevated TNF-*α* induces LRG1 secretion. LRG1 promotes angiogenesis coupling with *de novo* bone formation by recruiting MSCs in the subchondral bone of OA joints

In conclusion, during OA progression, the elevated TNF-*α* is the dominating proinflammatory cytokine that induces LRG1 secretion. LRG1 promotes angiogenesis coupling with *de novo* bone formation by recruiting MSCs in the subchondral bone of OA joints (Figure 6). LEN is an avenue for OA treatment.

## Materials and methods

### Mice

Male C57BL/6J mice were purchased from Shanghai SLAC Laboratory Animal Co., Ltd. The mice were maintained in a specific pathogen-free animal facility of the Institute of Health Sciences, Shanghai Institute for Biological Sciences and Shanghai Jiao Tong University School of Medicine.

Mice aged 8 weeks were anesthetized, and then transected the anterior cruciate ligament surgically to induce mechanical destabilized OA on the right knee. Sham operations were made a capsular incision on the independent mice. The operated mice were killed at 30 or 60 days after surgery (*n*=5 per group). For the therapeutic experiment 3 days before surgery, we injected LEN (Selleck, Houston, TX, USA; 50 mg per kg body weight) or the equivalent volume of vehicle (DMSO and PBS) intraperitoneally daily for 30 or 60 days. The mice were killed 30 or 60 days after surgery.

All the operations were performed under protocols approved by the Institutional Animal Care and Use Committee of the Institute of Health Sciences, Shanghai Institute for Biological Sciences and Shanghai Jiao Tong University School of Medicine.

### Histochemistry and immunohistochemistry

At the time of killing, the knee joints of mice were fixed in 4% paraformaldehyde, decalcified with 12.5% EDTA (pH 7.0) and embedded in paraffin. The knee joint sections (7 *μ*m) were stained with H&E and safranin O and fast green. For the immunostaining, the sections were incubated with primary antibodies to mouse nestin (1:800; Abcam, Cambridge, MA, USA), osterix (1:1000; Abcam), LRG1 (1:100; Abcam), CD31 (1:100; Abcam), TNF-*α* (1:100; Abcam) and MMP13 (1:200; Bioworld Technology) overnight at 4 °C. These samples were incubated with goat antirabbit secondary antibodies conjugated with horseradish peroxidase (HRP).

### Cell culture

The HUVECs and hBMMSCs were obtained from the Institute of Health Sciences, Shanghai Institute for Biological Sciences and Shanghai Jiao Tong University School of Medicine.

For cytokine stimulation analysis, HUVECs were cultured in a medium to full confluence, stimulated or not with different dosages of the indicated cytokines (IL-1*β*, TNF-*α*, IL-6 and IL-17; all from PeproTech, Rocky Hill, New Jersey, USA), and treated or not with different inhibitors: p38 MAPK inhibitor (SB203580, 10 *μ*M; Selleckchem, Houston, TX, USA) and NF-*κ*B inhibitor (BAY 11-7082, 10 *μ*M; Beyotime Co., Jiangsu, China).

### HUVEC tube-formation assay

Growth factor reduced Matrigel (BD Biosciences) was plated in 96-well culture plates and incubated at 37 °C to polymerize for 30 min. HUVECs (2 × 10^4^ cells/well) were seeded on polymerized Matrigel in the plates. The cells were cultured with medium alone or plus LRG1 (500 ng/ml; Abcam). After incubation at 37 °C for 4−6 h, we stained the cells with calcein and observed the tube formation by microscopy. The cumulative tube lengths were measured.

For the supernatant assay, HUVECs were transfected with small interfering RNA (siRNA; Shanghai GenePharma Co.,Ltd, Shanghai, China) for LRG1 (siLRG1) or scramble siRNA (siNC) and stimulated with TNF-*α* (30 ng/ml, PeproTech). Then the supernatants were collected after 3 days of stimulation. The supernatants combined with high-glucose DMEM medium were used to culture the HUVECs in the tube-formation assay. The siRNA for LRG1 was transfected using Lipofectamine 2000 (Invitrogen, Carlsbad CA, USA) as described by the manufacturer. The target sequence of LRG1 mRNA was 5′-GCAAUUAGAACGGCUACAU-3′, and that of scramble siRNA (siNC) was 5′-UUCUCCGAACGUGUCACGU-3′.

### The transwell assays of hBMMSCs

Cell migration was measured by using the 48-well Transwell plates (Millipore, Darmstadt, Germany) with 8 *μ*m pore filters. A total of 5.5 × 10^4^/well hBMMSCs were seeded in the upper chambers and then incubated them with medium alone or plus different concentrations of LRG1 in the lower chambers for 2−3 h at 37 °C in a 5% CO_2_-filled incubator. After incubation, the cells were fixed with 4% paraformaldehyde for 30 min. The cells on the upper surface of each filter were removed with cotton swabs. The cells that had migrated to the lower surface were stained with 0.5% crystal violet (Sigma-Aldrich, St. Louis, MO, USA) and photographed. Finally, the stained chambers were eluted with 33% acetic acid solution and quantified the eluent by using a TecanSafire2 microplate reader (Tecan, Durham, NC, USA) by absorbance at 570 nm. In the inhibitor assay, the hBMMSCs were preincubated with vehicle or p38 MAPK inhibitor (SB203580, 30 *μ*M, Selleckchem).

For the supernatant assay, the supernatants were collected as described above. The supernatants combined with high-glucose DMEM medium were added to the lower chambers.

### Osteogenic differentiation assay

The hBMMSCs were cultured in osteogenic medium containing 100 nM dexamethasone, 50 *μ*M ascorbic acid, and 10 mM *β*-glycerophosphate (all from Sigma-Aldrich). LRG1 (500 ng/ml; Abcam) was added. The medium was changed every 3 days. To assess the osteogenic differentiation, these cultures were stained with Alizarin Red S (Sigma-Aldrich). Finally, the calcium precipitates were dissolved in 0.1 N sodium hydroxide and quantified by using a TecanSafire2 microplate reader (Tecan) by absorbance at 548 nm.

### Cell proliferation assay

The hBMMSCs were seeded in 48-well plates for 24 h and then treated with LRG1 (500 ng/ml) for 0, 24 and 48 h. The cell viability was measured by using PrestoBlue Cell Viability Reagent (Thermo Scientific, Waltham, MA, USA) and quantified by using a TecanSafire2 microplate reader (Tecan) by excitation at 560nm and emission at 590 nm according to the manufacturer's instructions.

### RNA isolation and real-time polymerase chain reaction

The total RNA was isolated by using TRIZOL reagent (Invitrogen, Mulgrave, Australia). The RNA was reversely transcribed into cDNA by RevertAid Reverse Transcriptase (EP0442; Thermo Scientific) according to the manufacturer's instructions. Real-time polymerase chain reaction (RT-PCR) was performed with SYBRPremix EX Taq (Takara, Dalian, China) by using ViiATM 7 (Life Technologies, Carlsbad, CA, USA). Primers used in this study were the following: LRG1, 5′-GGACACCCTGGTATTGAAAGAAA-3′ and 5′-TAGCCGTTCTAATTGCAGCGG-3′.

### Western blot analysis

Western blot analysis was conducted by a previously described protocol.^44^ Proteins were separated by SDS–PAGE and transferred onto PVDF membranes (Millipore). Blots were probed with primary antibodies to LRG1 (1:500; Abcam), p38, phosphor-p38, p65, phosphor-p65 (1:1000; all from Cell Signaling Technology, Danvers, MA, USA), and GAPDH (1:5000; Kangcheng, Shanghai, China) overnight at 4 °C. These blots were then incubated with goat antirabbit secondary antibodies conjugated with HRP and visualized by using the enhanced chemiluminescence detection system (Millipore).

### Statistical analysis

Two-tailed Student's *t*-test and analysis of variance (ANOVA) were conducted to assess statistical significance. *P*<0.05 was considered statistically significant. Results are presented as the mean±S.D.
